# Analysis of Phenotypic and Genotypic Susceptibility to Clarithromycin and Amikacin of *Mycobacterium abscessus* Complex Strains Isolated from Cystic Fibrosis Patients

**DOI:** 10.3390/microorganisms11122897

**Published:** 2023-11-30

**Authors:** Juan Carlos Cao Yao, Jesús Navas Méndez, María Teresa Tórtola Fernández

**Affiliations:** 1 Department of Molecular Biology and Biomedicine, University of Cantabria, 39011 Santander, Spain; 2 Mycobacteria Unit, Clinical Laboratories, Microbiology Service, Hospital Universitari Vall d’Hebron, Universitat Autònoma de Barcelona; 08035 Barcelona, Spain

**Keywords:** *Mycobacterium*, clarithromycin, amikacin

## Abstract

*Mycobacterium abscessus* complex infections are ever on the rise. To curb their increasing evolution, we performed an in-depth study of 43 clinical isolates of cystic fibrosis patients obtained from 2009 to 2020. We identified their subspecies, uncovered their genotypic resistance profiles, characterised their antibiotic-resistant genes, and assessed their phenotypic antibiotic susceptibilities. The phenotypic and genotypic methods showed total agreement in terms of resistance to clarithromycin and amikacin. Of the 43 clinical strains, 28 belonged to *M. abscessus* subsp. *abscessus* (65.1%), 13 to *M. abscessus* subsp. *massiliense* (30.2%), and 2 to *M. abscessus* subsp. *bolletii* (4.6%). The resistant rates for clarithromycin and amikacin, the two main drugs against *M. abscessus* complex pulmonary infections, were 64.2% and 14.2%, respectively. We found three strains of *M. abscessus* subsp. *abscessus* that showed heteroresistance in the *rrl* and *rrs* genes, and these strains also presented double-resistance since they were macrolide- and aminoglycoside-resistant. *M. abscessus* subsp. *abscessus* showed a high minimum inhibitory concentration (MIC) and a resistant percentage larger than or equal to 88% to cefoxitin, ciprofloxacin, moxifloxacin, doxycycline, imipenem, and trimethoprim-sulfamethoxazole. These results show a panorama of the high resistance of *Mycobacterium abscessus* complex to current drugs for cystic fibrosis patients. Thus, other treatment methods are urgently needed.

## 1. Introduction

Cystic fibrosis (CF) is a rare fatal genetic disease that predominates in the northern European ethnicity. It is mainly caused by mutations in the cystic fibrosis transmembrane conductance regulator gene (CFTR), encoding for the membrane protein CFTR, which is mostly a chloride and bicarbonate transporter. Defects in the CFTR protein affect all epithelial cells in the body, as well as multiple organs. However, the pathology principally manifests in the respiratory tract since the abnormal osmotic imbalance of airway mucus thickens the mucus and impairs mucociliary clearance. This retained thick mucus constitutes an ideal microenvironment for infectious microorganisms [1,2,3].

The *Mycobacterium abscessus* complex (MABC) is one of the most important non-tuberculous mycobacteria (NTM) responsible for respiratory infections around the world. The lungs of cystic fibrosis (CF) patients provide a particularly favourable niche that fosters the colonisation and subsequent infection of many microorganisms [4]. Overall, NTM infections in CF patients have significantly escalated around the globe, rising from 3.3% to 22.6% in the last 20 years. *Mycobacterium avium* complex (MAC) and MABC form the highest proportion (95%) of NTM infections in CF patients, though MAC infections are decreasing as MABC infections are increasing [5]. The reported statistics, though, are bound to be lower than the reality, considering that NTM infections are commonly falsely diagnosed as other infections, such as tuberculosis, in developing countries.

MABC is a group of rapidly growing NTM comprising three different subspecies: *Mycobacterium abscessus* subsp. *abscessus*, *Mycobacterium abscessus* subsp. *massiliense,* and *Mycobacterium abscessus* subsp. *bolletii*. Identifying which subspecies is the cause of illness is crucial, as different subspecies differ in their levels of antibiotic resistance [6]. MABC’s high level of resistance to multiple drugs poses a great challenge for treatment. Clarithromycin, a macrolide, is one of the main drugs in the antibiotic regimen against lung infections caused by MABC [7,8]. Based on the functionality of the *erm*(41) gene, there are different CLA susceptibility patterns for the three MABC subspecies. Both *M. abscessus* subsp. *abscessus* and *M. abscessus* subsp. *bolletii* usually carry an inducible *erm*(41) gene T28 sequevar that confers inducible resistance to CLA [9]. A single-nucleotide mutation in the *erm*(41) gene, T28C (C28 sequevar), leads to a loss of methylase activity in *M. abscessus* subsp. *abscessus*, resulting in a phenotype that is susceptible to macrolides. *M. abscessus* subsp. *massiliense* has a non-functional *erm*(41) gene due to two characteristic deletions (bases 64–65 and 159–432), which render it susceptible to CLA [9,10,11]. Another important drug in the same regimen is amikacin (AMK) [7,8]. The first described case of high-level resistance to aminoglycosides was due to a single-point mutation within the *rrs* gene encoding for 16S rRNA [12].

Having more than one morphotype is a common property amongst NTM. MABC can exist with a smooth or rough morphotype. Smooth variants display glycopeptidolipids (GPLs) on the cell surface, which rough variants lack. The presence of GPLs is key to influencing host–pathogen interactions and allowing the aggregation of smooth bacteria into biofilms [13]. However, rough variants have also been observed to grow as biofilms in vitro under special conditions [14]. The smooth strains are considered wild-types, which become rough by mutation [15]. It has also been suggested that the rough morphotype is more virulent in humans [16].

The aim of this study is to investigate the subspecies, morphotypes, antibiotic susceptibility profiles, and molecular mechanisms of resistance to CLA and AMK in *Mycobacterium abscessus* complex strains isolated from CF patients.

## 2. Materials and Methods

### 2.1. Strains

**Figure 1:**
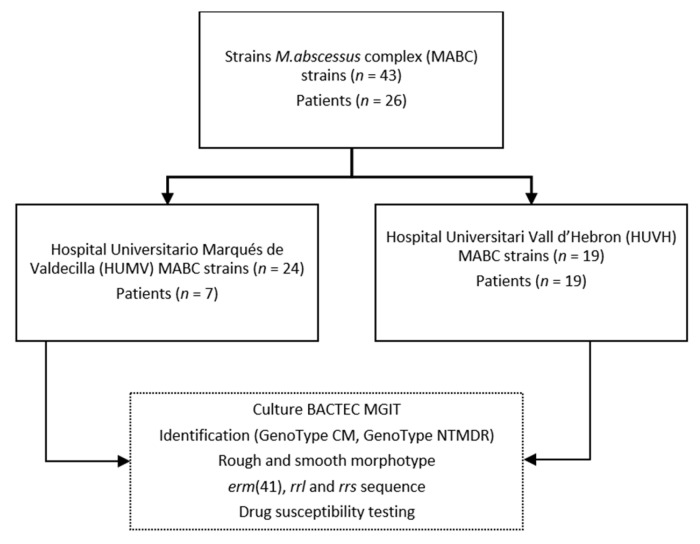
Schematic representation of the workflow followed in the study.

A total of 43 clinical strains were isolated from two clinical microbiology laboratories, of which 24 were provided by Hospital Universitario Marqués de Valdecilla (HUMV) and 19 were provided by Hospital Universitari Vall d’Hebron (HUVH). All isolates were harvested from 2009 to 2020 from sputum samples of 26 CF patients who met the criteria for a diagnosis of respiratory disease [17] and who had undergone prior antibiotic treatment (Figure 1).

### 2.2. Isolation and Identification

All isolates were obtained from primary isolation cultures in mycobacterial growth indicator tube (MGIT) liquid medium and detected using a BACTEC MGIT 960 instrument (Becton Dickinson, Franklin Lakes, NJ, USA).

Clinical strains were identified molecularly using a first screening with the GenoType Mycobacteria CM assay (Hain Lifescience GmbH, Nehren, Germany). The identification of subspecies and resistance genetic profiles was carried out through the GenoType NTM-DR molecular test (Hain Lifescience GmbH, Nehren, Germany).

### 2.3. Smooth and Rough Morphotypes

Colony morphology was assessed on Trypticase soy agar (BBL Microbiology Systems, Cockeysville, MD, USA).

### 2.4. Susceptibility Testing

For patients with more than one isolate, drug susceptibility testing (DST) was performed on the first available isolate and on those isolates that showed differences in terms of subspecies or genotype. DST was performed in cation-adjusted Mueller–Hinton medium using the broth microdilution method on Sensititre RAPMYCOI plates (Sensititre, Trek Diagnostic Systems, East Grinstead, UK), as recommended by the manufacturer. Plates were incubated at 30 °C with successive readings after 3, 5, 7, and 14 days. The initial reading time (IRT) was on day 3. If the growth-control well showed insufficient growth, the plate was re-incubated and read on days 5 and 7. The late reading time (LRT) was on day 14 of incubation, as described by Nash et al. [10]. Interpretations of the results were made according to the CLSI document M24-A2 [18].

### 2.5. erm(41), rrl, and rrs PCR for Sequencing

*erm*(41) detection was performed using the primers ERM1f (5′-CGCCAACGACGAGCAGCTCG-3′) and MC8-23R (5′-GACTTCCCCGCACCGATTCCAC-3′), as described by Bastian et al. and Nash et al., respectively [9,10]. *rrl* detection was performed using three primers: 18F (5′-AGTCGGGACCTAAGGCGAG-3′) and 21-R (5′-TTCCCGCTTAGATGCTTTCAG-3′) for PCR1, and 19-F (5′-GTAGCGAAATTCCTTGTCGG-3′) and 21-R for PCR 2. These primers were described by Meier et al. [19]. PCR1 and PCR2 were used for *rrl* detection and for sequencing, respectively. *rrs* detection was performed using the primers 1194F (5′-GAGGAAGGTGGGGATGACGT-3′) and 1525R (5′-AAGGAGGTGATCCAGCCGCA-3′). The numbers of the primers correspond to the position on the *E. coli* 16S rRNA gene [20]. PCRs were carried out as follows: 94 °C for 5 min, 40 cycles of 94 °C for 40 s, 62 °C for 50 s, 72 °C for 1 min, and 72 °C for 10 min. In the case of PCR1 for *rrl* detection, the extension time was 2 min rather than 1 min. Amplified DNA fragments were sequenced using the same primers as in PCR. Briefly, unincorporated nucleotides and primers were removed by ExoSAP-IT™ (Thermo Fisher Scientific, Waltham, MA, USA), and the gene targets were sequenced using a Big Dye Terminator Cycle Sequencing Ready Reaction kit (Applied Biosystems Inc., Foster City, CA, USA) in an ABI Prism 310 DNA sequencer (Applied Biosystems). Sequence alignment was performed using the programme MEGA 5. Homology analysis was performed by comparing the consensus sequences obtained for each isolate with those deposited in GenBank using the BLAST algorithm (Basic Local Alignment Search Tool, http://www.ncbi.nlm.nih.gov/BLAST, accessed on 10 July 2022). The *erm*(41) sequences of *M. abscessus* subsp. *abscessus* T28 sequevar ATCC 19977 (GenBank accession number HQ127365), *M. abscessus* subsp. *massiliense* CIP 108297 (GenBank HQ127368) and *M. abscessus* subsp. *bolletii* CIP 108541 (GenBank HQ127366) were used as references.

## 3. Results

### 3.1. Identification of Isolates

Out of the 43 MABC isolates, 28 belonged to *M. abscessus* subsp. *abscessus* (65.1%), 13 to *M. abscessus* subsp. *massiliense* (30.2%), and 2 to *M. abscessus* subsp. *bolletii* (4.6%). Of the first isolates from the 26 CF patients, 17 (65.3%) were *M*. *abscessus* subsp. *abscessus*, 7 (26.9%) were *M*. *abscessus* subsp. *massiliense*, and 2 (7.6%) were *M. abscessus* subsp. *bolletii* (Table 1).

### 3.2. Smooth and Rough Morphotypes

Of the first isolates from the 26 CF patients, 12 (46.1%) had rough morphotypes, 2 (7.6%) had smooth morphotypes, and 8 (30.7%) produced rough and smooth colonies simultaneously. The majority of rough morphotypes belonged to *M. abscessus* subsp. *abscessus* (75%) (Table 1).

From three patients, twenty isolates were obtained in total. For each patient, the first samples produced rough and smooth colonies simultaneously, but in the subsequent samples, only the rough morphotype was isolated (Table 2).

### 3.3. Genotyping of the erm and rrl Genes and Susceptibility Testing to Clarithromycin

There was a 100% concordance amongst DST, GenoType NTM-DR, and the sequencing of *erm*(41) and *rrl* genes. All of the *M. abscessus* subsp. *abscessus* strains had sequevar T28, except one strain, which had sequevar C28. For *M. abscessus* subsp. *massiliense*, we found two deletions within the *erm*(41) gene (nucleotides 64–65, and 276 nucleotides after nucleotide 158).

Drug susceptibility testing was performed for 28 strains (Table 3 and Table 4). The CLA resistance rate was 64.2% (18/28). According to the subspecies, CLA resistance was 72.2% (13/18) of *M. abscessus* subsp. *abscessus,* 37.5% (3/8) of *M. abscessus* subsp. *massiliense*, and 100% (2/2) of *M*. *abscessus* subsp. *bolletii*.

Three patterns of sensitivity were observed for CLA (Table 4). The first group was CLA-resistant after 72 h of incubation. Six *M. abscessus* subsp. *abscessus* strains and three *M. abscessus* subsp. *massiliense* strains presented these patterns, all of them having mutations in the *rrl* gene. These six samples presented acquired resistance to CLA, five presenting the A2058G point mutation in the *rrl* gene (two *M. abscessus* subsp. *abscessus* and three *M. abscessus* subsp. *massiliense*) and one (*M. abscessus* subsp. *abscessus*) presenting heteroresistance (concomitant infection with drug-resistant and drug-susceptible bacterial populations), where we observed the wild-type (WT) allele and two point-mutations, A2058G and A2059G, in the *rrl* gene. The second group comprised those isolates that were initially CLA-susceptible after 72 h, but demonstrated resistance at day 14 following prolonged incubation, implying inducible resistance. All of these isolates were *M. abscessus* subsp. *abscessus* (58.8%, 10/17) and all of them presented the T28 sequevar. The third group were isolates that remained susceptible after 14 days of incubation. Six strains showed this pattern: five *M. abscessus* subsp. *massiliense* T28 sequevar and one *M. abscessus* subsp. *abscessus* C28 sequevar.

### 3.4. Genotyping of the rrs Gene and Susceptibility Testing to Amikacin

There was a 100% concordance amongst DST, GenoType NTM-DR, and sequencing of the *rrs* gene. AMK was the most active antimicrobial against *M. abscessus* subsp. *abscessus* and *M. abscessus* subsp. *massiliense,* with susceptibility percentages of 76.4% and 75%, respectively (Table 4).

The percentage of resistance to amikacin was 14.2% (4/28). The subspecies of these four strains that presented mutations in the *rrs* gene were three *M. abscessus* subsp. *abscessus* and one *M. abscessus* subsp. *massiliense.* Two strains of *M. abscessus* subsp. *abscessus* presented heteroresistance, where we observed a wild-type (WT) allele and the point mutation A1408G. All the strains with mutations in the *rrs* gene also presented mutations in the *rrl* gene (4/28, 14.2%), and therefore were simultaneously resistant to AMK and CLA (Table 3).

### 3.5. Phenotypic Antibiotic Susceptibility Testing

The DST results of MABC are shown in Table 4. In general, the isolates were highly resistant to most of the agents tested, yielding similar results for the different studied subspecies. *M. abscessus* subsp. *abscessus* demonstrated high levels of resistance, with rates ≥ 88% to cefoxitina (FOX), ciprofloxacin (CIP), moxifloxacin (MXF), doxycycline (DOX), imipenem (IMP), and trimethoprim-sulfamethoxazole (SXT). *M. abscessus* subsp. *massiliense* was also highly resistant, but at a slightly lower rate: ≥75% to ciprofloxacin (CIP), moxifloxacin (MXF), doxycycline (DOX), imipenem (IMP), and trimethoprim-sulfamethoxazole (SXT).

For linezolid (LNZ), the numbers of sensitive and resistant strains were very similar for both *M. abscessus* subsp. *abscessus* and *M. abscessus* subsp. *massiliense. M. abscessus* subsp. *abscessus* had sensitivity and resistance rates to LNZ of 41.1% and 35.2%, respectively. For *M. abscessus* subsp. *massiliense*, the sensitivity and resistance rates to LNZ were both 37.5%.

There are no CSLI criteria for the interpretation of tigecycline (TGC), cefepime (FEP), amoxicillin-clavulanic acid (AUG), or ceftriaxone (AXO) [18]. Of all of them, TGC showed the best results. Using a breakpoint of ≥4 µg/mL as resistant, we obtained a TGC resistance rate of 28.5% (8/28), figuring in four isolates of *M. abscessus* subsp. *abscessus*, three of *M. abscessus* subsp. *massiliense*, and one of *M. abscessus* subsp. *bolletii.* The MABC resistance rates for FEP, AUG, and AXO were 96.4% (≥32), 96.4% (≥64/32), and 92.8% (≥64), respectively. The resistance rates were similar for the different MABC subspecies studied.

## 4. Discussion

Differentiation of the three MABC subspecies (*M. abscessus* subsp. *abscessus*, *M. abscessus* subsp. *Massiliense*, and *M. abscessus* subsp. *bolletii*) in routine diagnostic laboratories remains difficult. Due to horizontal gene transfer within the MABC, a single locus cannot be used to reliably determine or differentiate the subspecies within this complex. Therefore, the subspecies identification relies on the amplification and DNA sequencing of multiple genetic loci, including *hsp65*, *rpoB*, *secA1*, *sodA*, and the internal transcribed spacer (ITS) region between the 16S and 23S rRNA genes [21,22,23]. This method to identify MABC is complex and time-consuming. In routine diagnostic laboratories, it is more efficient to use a commercial technique for subspecies identification. We only used GenoType NTM-DR to that end, but other works which compared GenoType NTM-DR with other methods of identification showed 92–100% agreement [24,25]. Therefore, the GenoType NTM-DR is an accurate system for the identification of different subspecies of MABC. The *erm*(41) gene is not a subspecies-specific gene; therefore, *erm*(41) sequencing should not be used as the only technique to classify MABC subspecies [26]. However, by sequencing the *erm*(41) gene, we did obtain deletions in the characteristic positions of *M. abscessus* subsp. *massiliense* for the strains identified as such by GenoType NTM-DR.

In this study, the subspecies most frequently isolated was *M. abscessus* subsp. *abscessus* (65.3%), and the less frequently isolated were *M*. *abscessus* subsp. *massiliense* (26.9%) and *M. abscessus* subsp. *bolletii* (7.6%). When comparing our results with the literature, we observed that the proportions of the different MABC subspecies varied according to geographical distribution. The more predominant subspecies were *M. abscessus* subsp. *abscessus* and *M. abscessus* subsp. *massiliense*, responsible for >90% of MABC cases. In the United States and Europe, *M. abscessus* subsp. *abscessus* and *M. abscessus* subsp. *massiliense* account for 50–60% and 30–35%, respectively, of MABC pulmonary isolates from both CF and non-CF patients [6,23,27,28]. However, in other published works, in non-CF patients, these proportions are different. In a Spanish report, *M. abscessus* subsp. *abscessus* was the most frequently isolated subspecies (68.8%); the second was *M. abscessus* subsp. *bolletii* (25%); and *M. abscessus* subsp. *massiliense* (6.3%) was the least [26]. The results were completely different in South Korea, where the most prevalent subspecies were found to be *M. abscessus* subsp. *bolletii* and *M. abscessus* subsp. *massiliense*, constituting 58% and 55%, respectively [6,29].

MABC exhibits two different colony types: smooth and rough morphology [15]. Glycopeptidolipids (GPLs) are present in abundance in cell walls of the smooth morphotype, but in lower amounts in the rough morphotype [30]. GPLs play a role in environmental colonisation and are associated with sliding motility and biofilm formation. A marked reduction in the amount of GPL was correlated with cord formation, a property associated with mycobacterial virulence. *M. abscessus* was able to switch between smooth and rough morphologies, shifting between a colonising phenotype and a more virulent and invasive form [30]. Although, in our study, there were only few patients from whom we obtained several isolates over time, it is important to note that the first MABC isolates had colonies of mixed morphologies—smooth and rough—while in subsequent isolates only the rough morphology was observed. These results would indicate that at the beginning of lung colonisation, there were both smooth and rough colonies, and that later, colonies with rough morphology predominated, which are more virulent.

MABC strains with discrepant results between genotypic and phenotypic results for the *erm*(41) gene have been described. Different authors have recently documented strains of *M. abscessus* subsp. *abscessus* T28 sequevar that did not show inducible resistance to CLA, as they were susceptible to CLA after 14 days of incubation [29,31]. These strains had point mutations in codon 7 and in codon 67 of the *erm*(41) gene, resulting in a stop codon instead of arginine and in the loss of *erm*(41) gene function [29,31]. The results obtained in our study showed total consonance between the genotypic and phenotypic results. Our results of *erm*(41), *rrl*, and *rrs* sequencing are fully consistent with phenotypic CLA and AMK susceptibility. Inducible resistance and acquired resistance to CLA were observed when *erm*(41) was a T28 sequevar and when an *rrl* mutation was detected, respectively. Sensitivity to CLA was observed when *erm*(41) had a C28 sequevar or a deletion. These results are similar to those obtained by Bastian et al. [9]. Finally, resistance to AMK was observed when an *rrs* mutation was detected.

Our results show a very high percentage of resistance to all antibiotics studied. Amikacin was the only drug that showed an optimal percentage of sensitivity.

Of the 28 total MABC strains studied, 64.2% (18/28) were resistant to CLA regardless of the resistance mechanism. This resistance percentage amounted to 72.2% for *M. abscessus* subsp. *abscessus*. This is a very high resistance rate, very close to the 77% obtained by Bastian et al. [9], and much higher than the 13.6% obtained by Li et al. [32].

Acquired resistance to CLA is due to point mutations at positions 2058 and 2059 of the *rrl* gene. A previous study described *M. abscessus* isolates with acquired resistance to CLA that did not correlate with mutations in the *rrl* gene [33]. In our study, all the strains that showed acquired resistance (21.4%, 6/28) presented mutations in the *rrl* gene. The six strains with acquired resistance to CLA and mutations in the *rrl* gene consisted of three *M. abscessus* subsp. *abscessus* T28 sequevar and three *M. abscessus* subsp. *massiliense.* The detection of mutations in the *rrl* gene in *M. abscessus* subsp. *massiliense* was similar to other published works. The difference is that in our results, mutations in the *rrl* gene were also detected in *M. abscessus* subsp. *abscessus* sequevar T28, while in other works, *rrl* mutations in *M. abscessus* subsp. *abscessus* sequevar C28 were more frequent [9,34]. These results imply that the selection of resistant *rrl* mutants for the strains not expressing inducible resistance (37.5% of *M. abscessus* subsp. *massiliense*) was higher than for those strains that expressed inducible resistance (17.6% of *M. abscessus* subsp. *abscessus* sequevar T28).

The other important drug for the treatment of MAB infections is AMK [7,8]. The main mechanism of resistance to AMK is spontaneous mutations in the *rrs* gene that encode for the 16S rRNA, yielding a high level of resistance to AMK in patients with MABC isolates [12]. The percentage of acquired resistance to AMK was 14.2% for MABC and 16.6% for *M. abscessus* subsp. *abscessus*. Although AMK was the antibiotic with which the highest percentages of sensitivity were obtained, the percentage of AMK resistance of the MABC strains was 14.2%, a very high percentage when compared to that obtained by other authors, which ranged from 5.1% to 9.3% [31,32,35,36] This high percentage of AMK resistance could be due to the fact that the MABC strains were obtained from CF patients.

We found three strains that showed heteroresistance in the *rrl* and *rrs* genes detected by the GenoType NTM-DR and the sequencing of these genes. These heteroresistant strains also presented double-resistance since they were resistant to both macrolides and aminoglycosides. We did not find discrepant results between genotypic and phenotypic methods for susceptibility detection. It is worthy of remark that the percentage of MABC heteroresistant strains detected in the works in which GenoType NTM-DR was used ranged from 1.1% to 4% [24,37]. The highest percentage of heteroresistance (4%) was obtained from strains from CF patients [37]. Considering the low number of strains studied, the percentage of heteroresistance in our research was much higher, rising to 10.7% and 16.6% depending on whether we consider all MABC strains or only *M. abscessus* subsp. *abscessus*, respectively. These high percentages of resistance agree with the results obtained by Bastian et al., where CLA-resistant strains were more often isolated in CF patients [9]. These strains could indicate the presence of a much greater diversity of MABC populations in CF patients than in non-CF patients in whom MABC is also isolated. In a previous study in which whole-genome sequencing (WGS) was used, it was found that CF patients harboured multiple subpopulations, which were differentially abundant amongst sputum, lung, chest wound, and pleural fluid samples. Isolates of *M. abscessus* from sputum do not always reflect the diversity present within the patient, which can include subclones with different antimicrobial resistance profiles [38].

The percentage of resistance to the other drugs studied was also very high, whether they had CSLI criteria for interpretation (FOX, CIP, MXF, DOX, IMP, SXT) or not (TGC, FEP, AUG, AXO) [18], compared with previous publications. Only LNZ and TGC presented lower resistance percentages. Even so, they were also higher than those seen in the literature [31,39].

An interesting prospect in the treatment of patients with MABC isolates is synergy studies of different drugs. There have been reports of a better synergistic effect between CLA and MOX or TGC against *M. abscessus* subsp. *massiliense* than against *M. abscessus* subsp. *abscessus* [40]. These results corroborate the importance of the correct identification of MABC species or subspecies for better treatment outcomes. Furthermore, given the difficulty of treating isolates of *M. abscessus* subsp. *abscessus*, more research is needed in order to find the optimal treatment for its removal from clinical samples and to cure the patients.

A limitation of this study is the number of strains studied. A larger number of strains would have been advisable.

In conclusion, our data demonstrate that *M. abscessus* subsp. *abscessus* is the most common MABC subspecies in CF patients. The prevalence of resistance to almost all antibiotics tested, apart from AMK, was high. Due to the poor activity demonstrated by the antibiotics available for the treatment of infections caused by MABC, especially in CF patients, new drugs or other treatment methods are urgently needed.

## Figures and Tables

**Table 1:** *M. abscessus* subspecies and colony morphology of the first isolates obtained from the 26 CF patients.

	Colony Morphology				Total
	Rough	Smooth	Rough	Mixed	
*M*. *abscessus* subsp. *abscessus*	9	1	9	4	17
*M*. *abscessus* subsp. *massiliense*	1	1	1	4	7
*M. abscessus* subsp. *bolletii*	2	0	0	2	2

**Table 2:** Isolation chronology and colony morphotypes in CF patients with several MABC isolates.

		Isolation Chronology and Colony Morphotype			Total
		First Samples	Subsequent Samples		
		Mixed (Rough and Smooth)	Rough	Smooth	
Patient 1	*M*. *abscessus* subsp. *abscessus*	6	6	0	12
Patient 2	*M*. *abscessus* subsp. *massiliense*	2	1	0	3
Patient 3	*M*. *abscessus* subsp. *massiliense*	2	3	0	5

**Table 3:** *Mycobacterium abscessus* subspecies; susceptibility testing to clarithromycin and amikacin; and mutations in the *erm*, *rrl,* and *rrs* genes of the acquired-resistant isolates.

		Clarithromycin				Amikacin		
		S	I	R		S	I	R (*rrs* Gene Mutations)
				Inducible	Acquired (*rrl* Gene Mutations)			
*M. abscessus* subsp. *abscessus* (*n* = 18)	C28 sequevar	1				1		
	T28 sequevar		4	10	3 (WT + A2058G + A2059G, A2058C, and A2058C)	13	1	3 (A1408G, WT + A1408G, and WT + A1408G)
*M. abscessus* subsp. *massiliense* (*n* = 8)	T28 sequevar	5			3 (A2058C, A2058C, and A2058C)	6	1	1 (A1408G)
*M. abscessus* subsp. *bolletii* (*n* = 2)	T28 sequevar		1	1		2		

**Table 4:** Antimicrobial susceptibility testing of 28 *Mycobacterium abscessus* species. IRT, initial reading time; LRT, late reading time. S, susceptible; I, intermediately susceptible; R, resistant.

		Subsp. *abscessus*						Subsp. *massiliense* (*n* = 8) (%)			Subsp. *bolletii* (*n* = 2) (%)		
		C28 (*n* = 1) (%)			T28 (*n* = 17) (%)								
		S	I	R	S	I	R	S	I	R	S	I	R
AMK		1			13 (76.4)	1 (5.8)	3 (16.6)	6 (75)	1 (12.5)	1 (12.5)	2 (100)		
FOX		1				1 (5.8)	16 (94.1)	2 (25)	1 (12.5)	5 (62.5)		1 (50)	1 (50)
CIP				1	1 (5.8)		16 (94.1)		1 (12.5)	7 (87.5)			2 (100)
CLA	IRT	1					3 (16.6)	5 (62.5)		3 (37.5)	2 (100)		
	LRT	1			14 (82.3)		13 (76.4)	5 (62.5)		3 (37.5)		1 (50)	1 (50)
DOX				1		4 (23.6)	17 (100)		1 (12.5)	7 (87.5)			2 (100)
IMP				1			17 (100)			8 (100)			2 (100)
LNZ		1			7 (41.1)	4 (23.6)	6 (35.2)	3 (37.5)	2 (25)	3 (37.5)		1 (50)	1 (50)
MIN				1			17 (100)		1 (12.5)	7 (87.5)			2 (100)
MXF		1			1 (5.8)	1 (5.8)	15 (88.2)	2 (25)		6 (75)			2 (100)
SXT							17 (100)	2 (25)		6 (75)			2 (100)

## Data Availability

Data are contained within the article.

## References

[1] Edwards Q.T., Seibert D., Macri C., Covington C., Tilghman J. (2004). Assessing Ethnicity in Preconception Counseling: Genetics—What Nurse Practitioners Need to Know. J. Am. Acad. Nurse Pract..

[2] Elborn J.S. (2016). Cystic Fibrosis. Lancet.

[3] Kiedrowski M.R., Bomberger J.M. (2018). Viral-Bacterial Co-Infections in the Cystic Fibrosis Respiratory Tract. Front. Immunol..

[4] Strnad L., Winthrop K.L. (2018). Treatment of *Mycobacterium abscessus* Complex. Semin. Respir. Crit. Care Med..

[5] Janahi I.A., Rehman A., Sriramulu D. (2017). The Cystic Fibrosis Airway Microbiome and Pathogens. Progress in Understanding Cystic Fibrosis.

[6] Koh W., Jeon K., Lee N.Y., Kim B., Kook Y., Lee S., Park Y.K., Kim C.K., Shin S.J., Huitt G.A. (2011). Clinical Significance of Differentiation of *Mycobacterium massiliense* from *Mycobacterium abscessus*. Am. J. Respir. Crit. Care Med..

[7] Daley C.L., Iaccarino J.M., Lange C., Cambau E., Wallace R.J., Andrejak C., Böttger E.C., Brozek J., Griffith D.E., Guglielmetti L. (2020). Treatment of Nontuberculous Mycobacterial Pulmonary Disease: An Official ATS/ERS/ESCMID/IDSA Clinical Practice Guideline. Eur. Respir. J..

[8] Brown-Elliott B.A., Woods G.L. (2019). Antimycobacterial Susceptibility Testing of Nontuberculous Mycobacteria. J. Clin. Microbiol..

[9] Bastian S., Veziris N., Roux A.L., Brossier F., Gaillard J.L., Jarlier V., Cambau E. (2011). Assessment of Clarithromycin Susceptibility in Strains Belonging to the *Mycobacterium abscessus* Group by *erm*(41) and *rrl* Sequencing. Antimicrob. Agents Chemother..

[10] Nash K.A., Brown-Elliott A.B., Wallace R.J. (2009). A Novel Gene, *erm*(41) Confers Inducible Macrolide Resistance to Clinical Isolates of *Mycobacterium abscessus* but Is Absent from *Mycobacterium chelonae*. Antimicrob. Agents Chemother..

[11] Kim H.Y., Kim B.J., Kook Y., Yun Y.J., Shin J.H., Kim B.J., Kook Y.H. (2010). *Mycobacterium massiliense* Is Differentiated from *Mycobacterium abscessus* and *Mycobacterium bolletii* by Erythromycin Ribosome Methyltransferase Gene (*erm*) and Clarithromycin Susceptibility Patterns. Microbiol. Immunol..

[12] Prammananan T., Sander P., Brown B.A., Frischkorn K., Onyi G.O., Zhang Y., Böttger E.C., Wallace R.J. (1998). A Single 16S Ribosomal RNA Substitution Is Responsible for Resistance to Amikacin and Other 2-Deoxystreptamine Aminoglycosides in *Mycobacterium abscessus* and *Mycobacterium chelonae*. J. Infect. Dis..

[13] Johansen M.D., Herrmann J.-L., Kremer L. (2020). Non-Tuberculous Mycobacteria and the Rise of *Mycobacterium abscessus*. Nat. Rev. Microbiol..

[14] Ryan K., Byrd T.F. (2018). *Mycobacterium abscessus*: Shapeshifter of the Mycobacterial World. Front. Microbiol..

[15] Ridell M. (2015). *Mycobacterium abscessus*: An Environmental Mycobacteria Being a Human Pathogen. Int. J. Mycobacteriol..

[16] Catherinot E., Roux A.L., Macheras E., Hubert D., Matmar M., Dannhoffer L., Chinet T., Morand P., Poyart C., Heym B. (2009). Acute Respiratory Failure Involving an R Variant of *Mycobacterium abscessus*. J. Clin. Microbiol..

[17] Griffith D.E., Aksamit T., Brown-Elliott B.A., Catanzaro A., Daley C., Gordin F., Holland S.M., Horsburgh R., Huitt G., Iademarco M.F. (2007). An Official ATS/IDSA Statement: Diagnosis, Treatment, and Prevention of Nontuberculous Mycobacterial Diseases. Am. J. Respir. Crit. Care Med..

[18] Woods G.L. (2011). Susceptibility Testing of Mycobacteria, Nocardiae, and Other Aerobic Actinomycetes.

[19] Meier A., Kirschner P., Springer B., Steingrube V.A., Brown B.A., Wallace R.J., Bottger E.C. (1994). Identification of Mutations in 23S RRNA Gene of Clarithromycin-Resistant *Mycobacterium intracellulare*. Antimicrob. Agents Chemother..

[20] Maiwald M., Persing D.H., Tenover F.C., Versalovic J., Tang Y.W., Uger E.R., Relman D., White T.J. (2004). Broad-Range PCR for Detection and Identification of Bacteria. Molecular Microbiology: Diagnostic Principles and Practice.

[21] Macheras E., Roux A.L., Ripoll F., Sivadon-Tardy V., Gutierrez C., Gaillard J.L., Heym B. (2009). Inaccuracy of Single-Target Sequencing for Discriminating Species of the *Mycobacterium abscessus* Group. J. Clin. Microbiol..

[22] Nakanaga K., Sekizuka T., Fukano H., Sakakibara Y., Takeuchi F., Wada S., Ishii N., Makino M., Kuroda M., Hoshino Y. (2014). Discrimination of *Mycobacterium abscessus* subsp. *massiliense* from *Mycobacterium abscessus* subsp. *abscessus* in Clinical Isolates by Multiplex PCR. J. Clin. Microbiol..

[23] Zelazny A.M., Root J.M., Shea Y.R., Colombo R.E., Shamputa I.C., Stock F., Conlan S., McNulty S., Brown-Elliott B.A., Wallace R.J. (2009). Cohort Study of Molecular Identification and Typing of *Mycobacterium abscessus*, *Mycobacterium massiliense*, and *Mycobacterium bolletii*. J. Clin. Microbiol..

[24] Huh H.J., Kim S.Y., Shim H.J., Kim D.H., Yoo I.Y., Kang O.K., Ki C.S., Shin S.Y., Jhun B.W., Shin S.J. (2019). GenoType NTM-DR Performance Evaluation for Identification of *Mycobacterium avium* Complex and *Mycobacterium abscessus* and Determination of Clarithromycin and Amikacin Resistance. J. Clin. Microbiol..

[25] Kehrmann J., Kurt N., Rueger K., Bange F.-C., Buer J. (2016). GenoType NTM-DR for Identifying *Mycobacterium abscessus* Subspecies and Determining Molecular Resistance. J. Clin. Microbiol..

[26] Rubio M., March F., Garrigó M., Moreno C., Español M., Coll P. (2015). Inducible and Acquired Clarithromycin Resistance in the *Mycobacterium abscessus* Complex. PLoS ONE.

[27] Esther C.R., Henry M.M., Molina P.L., Leigh M.W. (2005). Nontuberculous Mycobacterial Infection in Young Children with Cystic Fibrosis. Pediatr. Pulmonol..

[28] Aitken M.L., Limaye A., Pottinger P., Whimbey E., Goss C.H., Tonelli M.R., Cangelosi G.A., Dirac M.A., Olivier K.N., Brown-Elliott B.A. (2012). Respiratory Outbreak of *Mycobacterium abscessus* Subspecies *massiliense* in a Lung Transplant and Cystic Fibrosis Center. Am. J. Respir. Crit. Care Med..

[29] Kim S.Y., Shin S.J., Jeong B.H., Koh W.J. (2016). Successful Antibiotic Treatment of Pulmonary Disease Caused by *Mycobacterium abscessus* Subsp. *abscessus* with C-to-T Mutation at Position 19 in *erm*(41) Gene: Case Report. BMC Infect. Dis..

[30] Howard S.T., Rhoades E., Recht J., Pang X., Alsup A., Kolter R., Lyons C.R., Byrd T.F. (2006). Spontaneous Reversion of *Mycobacterium abscessus* from a Smooth to a Rough Morphotype Is Associated with Reduced Expression of Glycopeptidolipid and Reacquisition of an Invasive Phenotype. Microbiology.

[31] Jong B.-E., Wu T.-S., Chen N.-Y., Yang C.-H., Shu C.-C., Wang L.-S., Wu T.-L., Lu J.-J., Chiu C.-H., Lai H.-C. (2022). Impact on Macrolide Resistance of Genetic Diversity of *Mycobacterium abscessus* Species. Microbiol. Spectr..

[32] Li Y.M., Tong X.L., Xu H.T., Ju Y., Cai M., Wang C. (2016). Prevalence and Antimicrobial Susceptibility of *Mycobacterium abscessus* in a General Hospital, China. Biomed. Environ. Sci..

[33] Carneiro M.D., Nunes L.D., David S.M., Barth A.L. (2017). Lack of Association between *rrl* and *erm*(41) Mutations and Clarithromycin Resistance in *Mycobacterium abscessus* Complex. Mem. Inst. Oswaldo Cruz.

[34] Mougari F., Bouziane F., Crockett F., Nessar R., Veziris N., Sapriel G., Raskine L., Cambau E. (2017). Selection of Resistance to Clarithromycin in *Mycobacterium abscessus* Subspecies. Antimicrob. Agents Chemother..

[35] Zhang Z., Wang W., Wang Y., Xue Z., Li S., Pang Y. (2022). Inducible Resistance to Amikacin in *Mycobacterium abscessus* Isolated in Beijing, China. Infect. Drug Resist..

[36] Ananta P., Kham-ngam I., Chetchotisakd P., Chaimanee P., Reechaipichitkul W., Namwat W., Lulitanond V., Faksri K. (2018). Analysis of Drug-Susceptibility Patterns and Gene Sequences Associated with Clarithromycin and Amikacin Resistance in Serial *Mycobacterium abscessus* Isolates from Clinical Specimens from Northeast Thailand. PLoS ONE.

[37] Mougari F., Loiseau J., Veziris N., Bernard C., Bercot B., Sougakoff W., Jarlier V., Raskine L., Cambau E., Aubry A. (2017). Evaluation of the New GenoType NTM-DR Kit for the Molecular Detection of Antimicrobial Resistance in Non-Tuberculous Mycobacteria. J. Antimicrob. Chemother..

[38] Shaw L.P., Doyle R.M., Kavaliunaite E., Spencer H., Balloux F., Dixon G., Harris K.A. (2019). Children with Cystic Fibrosis Are Infected with Multiple Subpopulations of *Mycobacterium abscessus* with Different Antimicrobial Resistance Profiles. Clin. Infect. Dis..

[39] Chua K.Y.L., Bustamante A., Jelfs P., Chen S.C.-A., Sintchenko V. (2015). Antibiotic Susceptibility of Diverse *Mycobacterium abscessus* Complex Strains in New South Wales, Australia. Pathology.

[40] Zhang Z., Lu J., Liu M., Wang Y., Zhao Y., Pang Y. (2017). In Vitro Activity of Clarithromycin in Combination with Other Antimicrobial Agents against *Mycobacterium abscessus* and *Mycobacterium massiliense*. Int. J. Antimicrob. Agents.

